# Cost and effectiveness of autologous haematopoietic stem cell transplantation and high-efficacy disease-modifying therapies in relapsing–remitting multiple sclerosis

**DOI:** 10.1007/s10072-024-07308-y

**Published:** 2024-01-26

**Authors:** Alice Mariottini, Chiara Nozzoli, Ilaria Carli, Filippo Landi, Valentina Gigli, Anna Maria Repice, Alessandra Ipponi, Michele Cecchi, Riccardo Boncompagni, Riccardo Saccardi, Luca Massacesi

**Affiliations:** 1 Department of Neurosciences, Drug and Child Health, University of Florence, Florence, Italy; 2 Department of Neurology 2 and Tuscan Region Multiple Sclerosis Referral Centre, Careggi University Hospital, Florence, Italy; 3 Cell Therapy and Transfusion Medicine Unit, Careggi University Hospital, Florence, Italy; 4 Hospital Management, UOC Controllo Direzionale, Careggi University Hospital, Florence, Italy; 5 Hospital Pharmacy, Careggi University Hospital, Florence, Italy

**Keywords:** Hematopoietic stem cell transplantation, Multiple sclerosis, Healthcare costs, Disease-modifying therapies, Disability, Treatment outcome

## Abstract

**Background:**

Autologous haematopoietic stem cell transplantation (AHSCT) is a highly effective one-off treatment for relapsing–remitting multiple sclerosis (RR-MS), potentially representing an optimal front-loading strategy for costs.

**Objective:**

Exploring cost/effectiveness of AHSCT and high-efficacy disease-modifying treatments (HE-DMTs) in RR-MS, estimating costs at our centre in Italy, where National Health Service (NHS) provides universal health coverage.

**Methods:**

Costs (including drugs, inpatient/outpatient management) for treatment with AHSCT and HE-DMTs were calculated as NHS expenditures over 2- and 5-year periods. Cost-effectiveness for each treatment was estimated as “cost needed to treat” (CNT), i.e. expense to prevent relapses, progression, or disease activity (NEDA) in one patient over *n*-years, retrieving outcomes from published studies.

**Results:**

Costs of AHSCT and HE-DMTs were similar over 2 years, whereas AHSCT was cheaper than most HE-DMTs over 5 years (€46 600 vs €93 800, respectively). When estimating cost-effectiveness of treatments, over 2 years, mean CNT of HE-DMTs for NEDA was twofold that of AHSCT, whereas it was similar for relapses and disability. Differences in CNT were remarkable over 5 years, especially for NEDA, being mean CNT of HE-DMTs €382 800 vs €74 900 for AHSCT.

**Conclusions:**

AHSCT may be highly cost-effective in selected aggressive RR-MS. Besides priceless benefits for treated individuals, cost-savings generated by AHSCT may contribute to improving healthcare assistance at a population level.

## Background

Autologous haematopoietic stem cell transplantation (AHSCT) is considered a treatment option for relapsing–remitting (RR) multiple sclerosis (MS) refractory to conventional disease-modifying treatments (DMTs) [1], proved to be superior to DMTs (ocrelizumab and alemtuzumab excluded) by one randomized clinical trial [2]. AHSCT eradicates and reconstitutes the haematopoietic system of the treated individual, promoting the restoration of a new immune tolerance [3] and determining a complete suppression of new focal inflammatory activity in most of the treated cases [4].

AHSCT is administered as a one-off treatment and does not require any maintenance therapies thereafter, unless a disease reactivation is observed. On the other hand, most of the approved DMTs require chronic administration, which is associated with considerable yearly expenses for National Health Services (NHS).

The cost-effectiveness of AHSCT was previously explored in people affected by secondary-progressive (SP-) MS, where AHSCT appeared to be cost-effective compared to mitoxantrone in two out of three scenarios evaluated [5]. More recently, two analyses of costs were performed in the USA [6] and Poland [7], both showing that AHSCT could generate remarkable cost savings in the mid-long term.

As differences in healthcare systems and DMTs availability across countries might limit the generalisation of such findings, in the present study, the NHS expense for myeloablative intermediate-intensity AHSCT was estimated and compared to costs for treatment with high-efficacy (HE-) DMTs in Italy, where universal health coverage is provided.

## Materials and methods

The study was performed at the University Hospital of Careggi in Florence, Italy.

Total expenses for treating one RR-MS patient with AHSCT or HE-DMTs were estimated as ideal “standard costs”, i.e. considering routine instrumental examinations and blood tests performed according to local protocols, adopted by the centre in clinical practice.

As DMTs may be administered either with chronic or pulsed schedule (usually over 2 years), and AHSCT is a one-off therapy, the cumulative cost of treatment was estimated considering both 2- and 5-year periods. The 5-year period was chosen as it represents the timeframe adopted by most open-label extension studies of approved DMTs.

### AHSCT

Costs were estimated for AHSCT with the intermediate-intensity conditioning regimen BEAM/ATG [1], the protocol adopted by our centre. Briefly, mobilisation of haematopoietic stem cells (HSCs) from the bone marrow is obtained using the association of cyclophosphamide (4 g/m^2^ body surface area [BSA]) in two doses on the same day followed by daily granulocyte colony-stimulating factor starting at day + 5 (G-CSF, 10 μg/kg per day), until completion of the harvest. Peripheral blood haematopoietic stem cells (PBSCs) are collected with leukapheresis and cryopreserved until the transplant. The conditioning regimen encompasses BCNU (Carmustine) 300 mg/m^2^ BSA on day − 6, ARA-C (Cytosine-Arabinoside) 200 mg/m^2^/day and VP-16 (Etoposide) 200 mg/m^2^/day from day − 5 to day − 2, and Melphalan 140 mg/m^2^ on day − 1; rabbit anti-thymocyte globulin (ATG, Thymoglobulin, Sanofi) is added at a dose of 3.75 mg/kg/day on day + 1 and + 2 (total dose 7.5 mg/Kg).

Costs for treatment with AHSCT were estimated including all the followings: eligibility screening; chemotherapy drugs, symptomatic and supportive treatments, antimicrobial therapy (including antimicrobial prophylaxis administered after hospital discharge); inpatient stay (expense for health workers and overheads included); and blood tests and examinations performed as post-treatment monitoring, including seriate polymerase chain reaction (PCR) testing for early detection of Epstein-Barr virus (EBV) and cytomegalovirus (CMV) reactivations.

Procedures for preservation of fertility, i.e. oocyte and sperm cryopreservation, are currently discussed and offered to all the patients eligible for AHSCT at our centre. Costs related to these procedures were not included in the “standard cost” of AHSCT because fertility preservation is not performed routinely in all cases, as access to the procedure is ultimately determined by patients’ choice and family planning (e.g. patients who have already fulfilled before AHSCT their desire for parenthood may decide not to receive fertility preservation). Nonetheless, given the relevance of fertility issues after AHSCT, costs for procedures for fertility preservation were calculated, and reported separately from the “standard cost” and main CNT analyses. The costs were provided separately for AHSCT including either oocyte or sperm cryopreservation, due to the different price ranges of these procedures.

A “standard” duration of inpatient stay was estimated from the average inpatient stay observed for the procedures performed over the last 5 years.

### HE-DMTs

As comparative treatments, the following HE-DMTs that were licensed in Italy up to June 2022 as “second line DMTs” for the treatment of RR-MS were included: natalizumab, fingolimod, alemtuzumab, ocrelizumab, and cladribine.

For HE-DMTs with continuative administration (natalizumab, fingolimod, and ocrelizumab), cumulative costs for each year of treatment were estimated including all the followings: DMT price for a 1-year course of treatment, costs for routinely blood and instrumental tests performed for treatment monitoring over 1 year, and costs related to DMT administration in inpatient setting, where applicable (natalizumab, ocrelizumab, and first dose of fingolimod). The cumulative expense for a 2- or 5-year course of treatment was then calculated by multiplying the yearly cost (obtained as described above) by 2 or 5, respectively, and adding the cost of the eligibility screening, which was performed only once (i.e. before treatment commencement).

For HE-DMTs with pulsed administration (alemtuzumab and cladribine), DMT cost was estimated for a standard 2-year course of treatment. Accordingly,  in the 5-year period, expenses for years three to five included only the costs of treatment monitoring.

Costs of HE-DMTs and ancillary drugs were extrapolated from contract prices provided by the Hospital Pharmacy; the expense for blood tests and instrumental examinations were calculated from the “Nomenclatore” of the Careggi University Hospital, i.e. a document listing the healthcare services and respective costs provided by the hospital. Expenses for consumables and procedures performed in inpatient setting were also estimated, including costs of health workers and overheads. All the amounts are expressed in euros (€).

### Cost needed to treat

As different HE-DMTs are associated with different rates of survival free from disease activity over a definite period, a rough comparison of costs required to achieve similar effectiveness at a population level was performed as a standard “cost needed to treat” (CNT).

CNT was calculated for AHSCT and each HE-DMT as follows: number of patients needed to treat with therapy “X” to prevent MS disease activity (as defined below) in one patient in the timeframe selected (2 or 5 years), multiplied by the cost of the treatment “X” over the same timeframe. As an example, if MS activity-free survival was 20% at 2 years with treatment “X”, and the cost of a 2-year course with this treatment was €20 000, CNT would be 5 (number of patients needed to treat with therapy “X” to achieve freedom from MS activity in one case over 2 years) × €20 000 (cost of treatment “X” over a 2-year period) = €100 000.

All the following were adopted as indicators of MS activity: relapse-free survival (RFS), progression-free survival (PFS), and no evidence of disease activity (NEDA) survival, the latter defined as the absence of all the followings: relapses, disability worsening, and new inflammatory activity at brain magnetic resonance imaging (MRI).

Data for AHSCT were extrapolated from the widest cohorts of RRMS patients treated with BEAM/ATG regimen published to date [8, 9].

For HE-DMTs, data on efficacy outcomes were retrieved from pivotal trials or, where not available, from open-label extension studies or observational registry studies. In detail, for each HE-DMT data were extracted from the following studies: natalizumab, AFFIRM [10, 11], and TOP studies [12]; fingolimod, FREEDOMS [13, 14], and LONGTERMS [15]; alemtuzumab, CARE-MS II [16] and extension [17] studies; ocrelizumab, OPERA I–II [18] and extension [19] studies; cladribine, CLARITY [20], and extension [21, 22] studies.

However, the CNT analyses should be taken with caution as they derive from indirect comparisons of studies that included heterogeneous patient populations. Furthermore, data at year 5 were not available for all the HE-DMTs analysed.

### Statistical methods

Cumulative cost for each treatment was obtained by summation of the cost of each item considered. For treatments administered with a dose dependent on patients’ weight (cladribine and ATG) or BSA (drugs in the BEAM protocol), cumulative dosage was calculated using an ideal weight and BSA of 65 kg and 1.75 m^2^, respectively. Mean cost of all the HE-DMTs included, or HE-DMTs with pulsed/continuative administration was also calculated.

## Results

### “Standard cost” of treatment with AHSCT and HE-DMTs

The estimated “standard cost” of AHSCT was €44 500, including costs for pre-treatment screening tests, drugs administered and inpatient care during the AHSCT procedure, and outpatient care and monitoring over the first year (as detailed in the methods section). The expense for drugs in the BEAM/ATG protocol was about 10% of the total cost, and most of the expense was due to inpatient care. Costs of oocyte and sperm cryopreservation were estimated as roughly €1800 and €500, respectively, thus representing about 4% and 1% of the “standard cost” of AHSCT, respectively. When including procedures for preservation of fertility in the AHSCT pathway, AHSCT cost was estimated to be €46 300 and €45 000 for females and males, respectively.

For HE-DMTs with chronic administration (natalizumab, fingolimod, ocrelizumab), yearly expenses ranged from roughly €21 100 to €28 300. The cost of a 2-year course of treatment with cladribine or alemtuzumab was roughly €36 000 and €62 700, respectively. Costs due to inpatient management were absent for cladribine and marginal for fingolimod, whereas they represented 20–25% of the total expense for the remaining HE-DMTs.

### Cumulative expense for treatment over 2- and 5-year periods

**Fig. 1:**
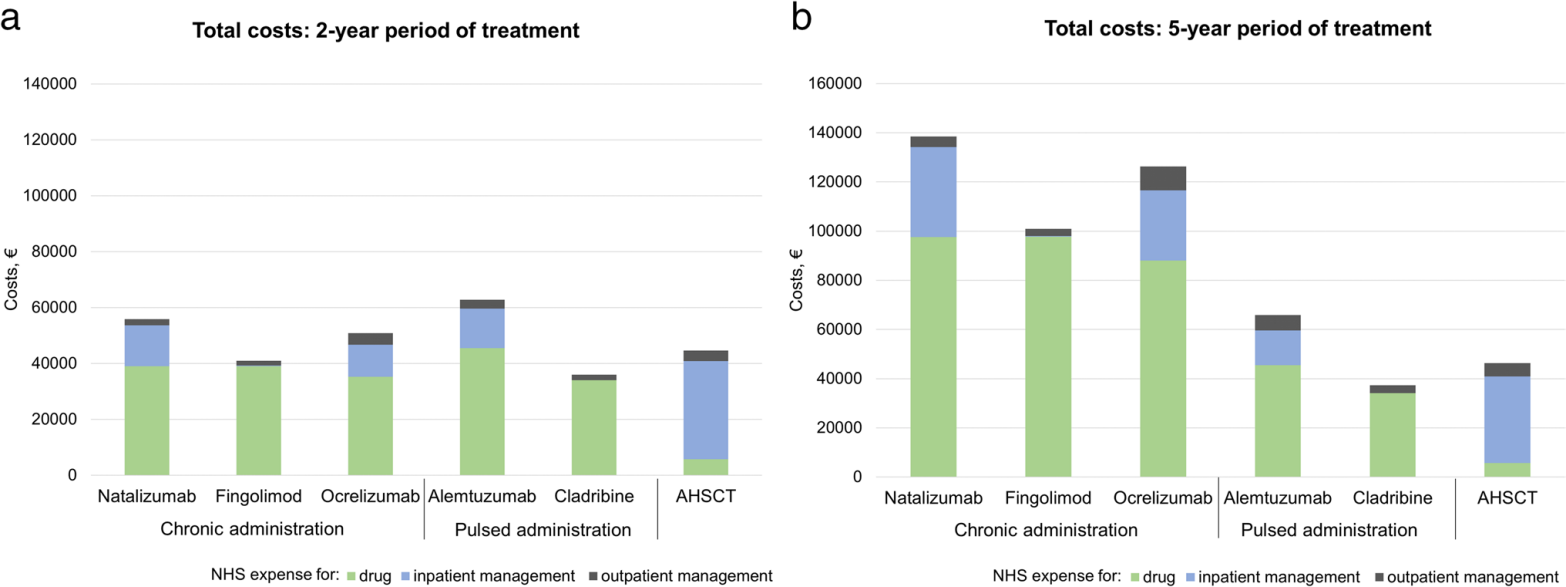
Cumulative cost for a 2-year period (**a**) and a 5-year period (**b**) of treatment with AHSCT and high-efficacy DMTs with chronic or pulsed administration schedule. Expenses for inpatient management include costs for consumables, tests, ancillary drugs, healthcare workers, and overheads. Expenses for outpatient management include costs of standard treatment monitoring. For DMTs with a pulsed administration schedule, a 2-year course of treatment was considered, with costs of monitoring only over the subsequent three years. Over a 2-year period (**a**), average costs are similar across treatments. Over a 5-year period (**b**), expenses for DMTs with a chronic administration schedule are higher than those for pulsed therapies and AHSCT (mean €121 900, €51 600 and €46 600, respectively)

When considering a 2-year period of treatment (Fig. 1a), AHSCT cost was roughly €45 000, and HE-DMTs ranged from roughly €36 000 to €62 700. Compared to AHSCT, cladribine and fingolimod were cheaper (− 20% and − 8%, respectively), whereas the remaining HE-DMTs were more expensive by 14 to 40%.

Over﻿ a 5-year period (Fig. 1b), AHSCT cost was roughly €46 600, and HE-DMTs ranged from roughly €37 200 to €138 500. Compared to AHSCT, cladribine only was cheaper (− 20%), whereas costs for the remaining HE-DMTs were 42 to 200% higher. Over this timeframe, AHSCT and HE-DMTs with pulsed administration were cheaper than HE-DMTs with chronic administration schedules, with a mean cost of €51 600 and €121 900, respectively.

When including oocyte/sperm cryopreservation in the AHSCT procedure, the estimated cost of AHSCT over a 2- and 5-year period was approximately €46 800/€45 500 and €48 400/€47 100, respectively.

### Cost needed to treat

**Fig. 2:**
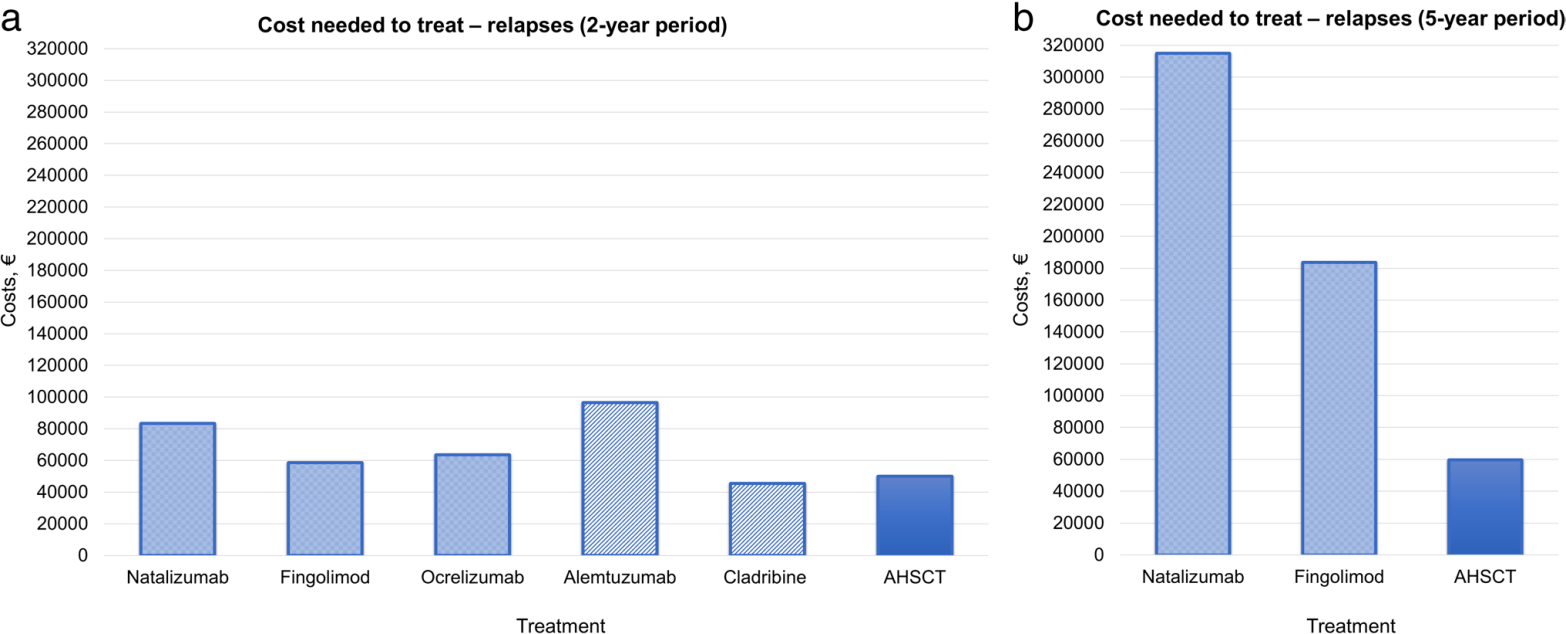
Cost needed to treat (CNT) of AHSCT and HE-DMTs to obtain one patient free from relapse activity over a 2-year (**a**) and 5-year period (**b**). Estimates of relapse-free survival (RFS) for AHSCT and HE-DMTs are extrapolated from prospective or cohort studies on BEAM-based AHSCT and RCTs and extension studies, respectively (details in the “Materials and methods” section)

**Fig. 3:**
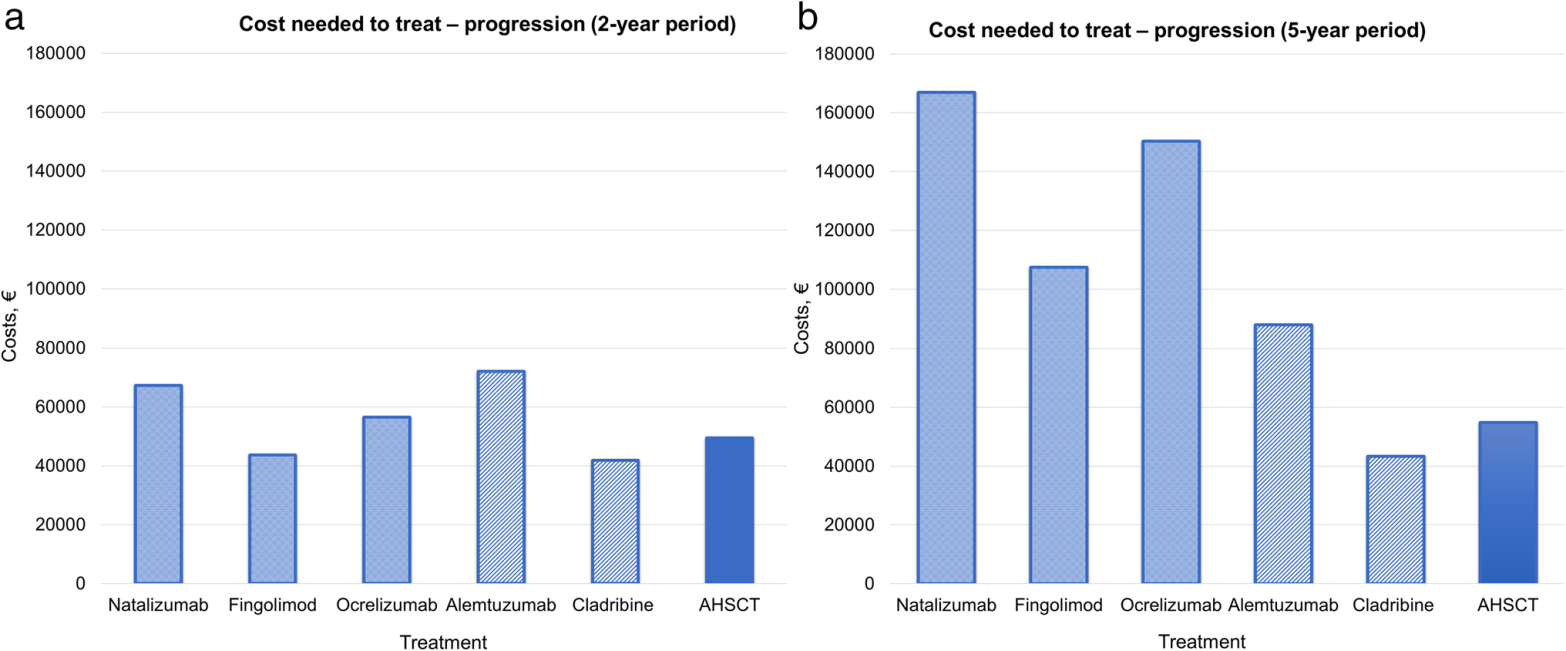
Cost needed to treat (CNT) of AHSCT and HE-DMTs to obtain one patient free from disability progression over a 2-year (**a**) and 5-year period (**b**). Estimates of progression-free survival (PFS) for AHSCT and HE-DMTs are extrapolated from prospective or cohort studies on BEAM-based AHSCT and RCTs and extension studies, respectively (details in the “Materials and methods” section)

**Fig. 4:**
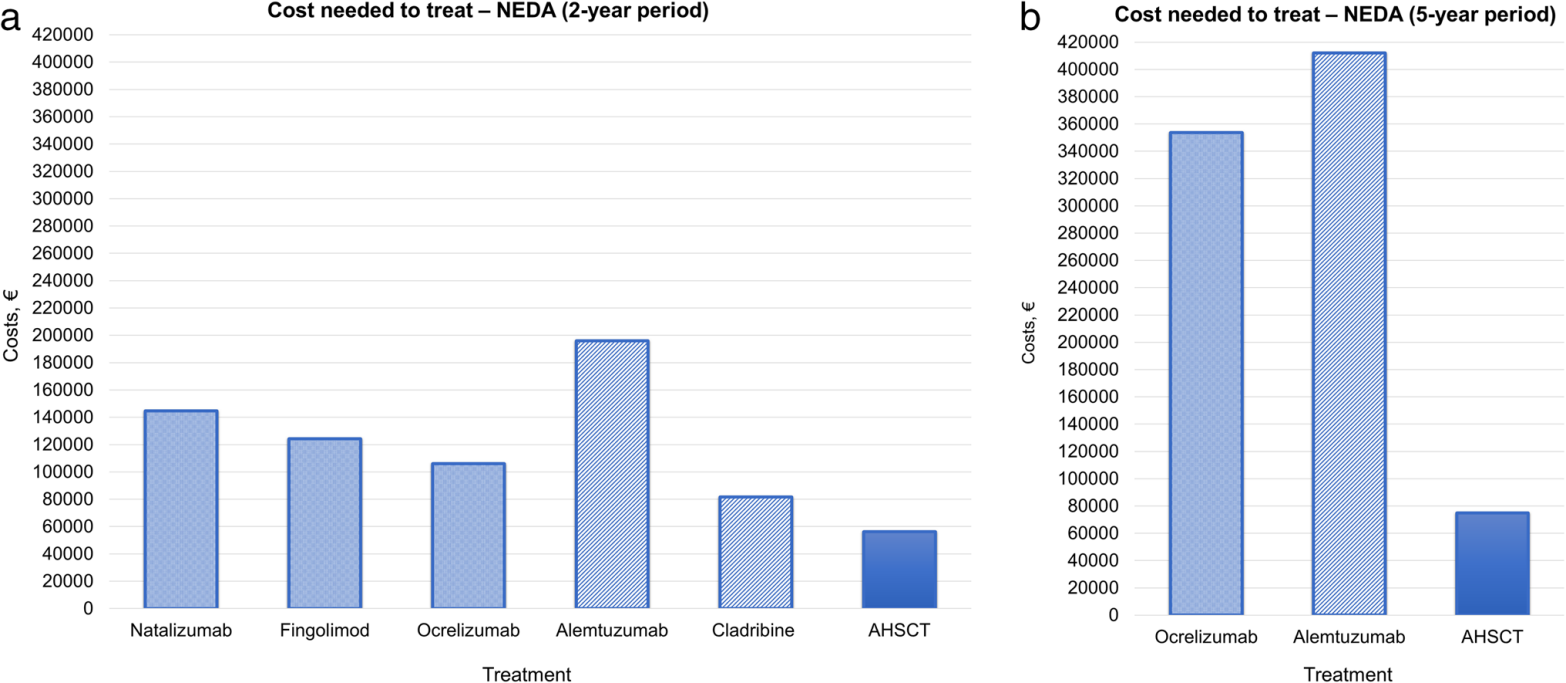
Cost needed to treat (CNT) of AHSCT and HE-DMTs to obtain one patient free from clinical and radiological disease activity over a 2-year (**a**) and 5-year period (**b**). Estimates of no-evidence of disease activity (NEDA) survival for AHSCT and HE-DMTs are extrapolated from prospective or cohort studies on BEAM-based AHSCT and RCTs and extension studies, respectively (details in the “Materials and methods” section)

Over a 2-year period, CNT for RFS (Fig. 2a) and PFS (Fig. 3a) was overall similar across treatments, with few exceptions. The mean CNT of HE-DMTs for RFS and PFS was €69 500 and €56 300, respectively, whereas for AHSCT, it was €50 000 and €49 400, respectively. Differences were more prominent when considering CNT for maintaining NEDA status (Fig. 4a), where HE-DMTs were up to threefold more expensive than AHSCT, with a mean CNT of €130 600 and €56 200, respectively.

Compared﻿ to the 2-year period, differences across treatments were more evident over 5 years for RFS (Fig. 2b) and NEDA (Fig. 4b): CNT for RFS was from threefold to fivefold higher for HE-DMTs (mean €249 300) compared to AHSCT (€59 700), and it was fivefold higher for NEDA, with a mean CNT of €382 800 for HE-DMTs compared to €74 900 for AHSCT. At year 5, the mean CNT for PFS (Fig. 3b) was €111 200 for HE-DMTs and €54 800 for AHSCT.

When including oocyte or sperm cryopreservation in the AHSCT procedure, CNT of AHSCT increased by roughly 4% or 1% compared to the above-reported estimates, respectively. In detail, CNT for RFS, PFS, and NEDA over 2 years inclusive of oocyte/sperm cryopreservation was €52 000/€50 600, €51 500/€50 000, and €58 500/€56 900, respectively. Over 5 years, CNT for RFS, PFS and NEDA (oocyte/sperm cryopreservation included) was €62 100/€60 400, €57 000/€ 55500, and € 77900/€ 75800, respectively. Such increase did not substantially affect the gap in CNT between AHSCT and HE-DMTs observed in long-term outcomes.

## Discussion

The approval of HE-DMTs has radically changed the natural history of MS, contributing to the observed improvement in long-term outcomes and patients’ quality of life [23]. As the early use of HE-DMTs may offer advantages over moderate-efficacy DMTs [24, 25], early escalation and “top-down” strategies have been increasingly used, with a potential economic burden for NHS due to expensive treatment-related costs and unequal availability of HE-DMTs across countries [26]. Despite the high costs of HE-DMTs, their early use could allow reducing NHS expenditure in the long-term with “front-loading” of the costs, i.e. investing more resources early during MS course to stabilise the disease and reduce long-term costs related to disability accrual and potential complications [27]. As events of progression and relapse were estimated to increase mean quarterly societal economic costs by 29% and 56%, respectively [28], the reduction of the societal economic burden of MS is likely proportional to the effectiveness of the treatment used. In this respect, indirect comparisons suggest that AHSCT may induce rates of NEDA remarkably higher than DMTs [2, 29]. Even if head-to-head comparisons with HE-DMTs are lacking, possible superior effectiveness of AHSCT was suggested over natalizumab [2, 30], fingolimod [30], and alemtuzumab [31]. Indeed, disease burden and accumulated disability are usually higher in the published experiences on AHSCT than in patients included in Registration Studies, therefore providing an unfavourable outcome scenario in AHSCT.

Two studies previously analysed the costs of AHSCT in RR-MS in two different healthcare systems [6, 7], reporting costs of AHSCT ranging from less than €20 000 in Poland to an average of $85 184 (range $70 635 to $120 260) in the USA. In our study, the “standard cost” of AHSCT and related monitoring over the first year was estimated at around €44 000 (up to € 46 300 when including procedures for fertility preservation), and it was lower than the costs of most HE-DMTs over 5 years, as expected due to its “one-off” administration. Our “standard cost” was estimated for AHSCT with the BEAM/ATG regimen; therefore, it could vary when using other conditioning protocols, such as Cy/ATG, which is widely used in Europe and the USA (and adopted in the study by Burt et al. [6]). Nonetheless, variations in the conditioning protocol would plausibly affect marginally the cumulative expense for AHSCT, as this was mostly due to costs related to the procedure rather than to chemotherapy. On the contrary, costs of the immunomodulant drug were the main component of the expense for treatment with HE-DMTs, although inpatient management accounted for up to one-quarter of the total expense for therapies requiring chronic administration in inpatient setting. Both these expenditure items in HE-DMTs treatment may indeed be lowered in the future. First, the expense for immunomodulatory treatments might remarkably decrease with the use of generic drugs and biosimilars instead of branded drugs, which may plausibly occur in the near future [32]. Second, the recent approval of HE-DMTs that can be administered subcutaneously at home or the hospital facility with a short inpatient stay [33, 34] may generate further cost savings by avoiding or reducing expenses for inpatient management [35] and indirect costs due to loss of working days.

Besides raw NHS expenses, a simplified cost-effectiveness analysis was performed, trying to account for differences in effectiveness across treatments. A cost needed to treat (CNT) was therefore estimated to assume that each treatment exerted a similar effect on the target population, deriving raw data on efficacy outcomes from published trials. When considering analyses of CNT, costs were similar over the short term except for achieving NEDA status, where HE-DMTs were up to threefold more expensive than AHSCT. As expected, the difference in costs increased remarkably over 5 years, with a mean CNT to obtain NEDA fivefold higher with HE-DMTs compared to AHSCT. Such differences may be even more conspicuous at longer follow-up, assuming that NEDA status might be maintained by a relevant proportion of RR-MS patients up to (and plausibly even after) 10 years following AHSCT. Supporting this scenario, NEDA survival rates in RR-MS ranged from 40.5 [8] to 70% [36] at 10 years after AHSCT in published studies exploring long-term outcomes.

Based on these observations, AHSCT may represent an optimal front-loading strategy of costs in RR-MS, especially in cases bearing poor prognostic factors. Besides lower treatment-cost to prevent long-term disability accrual (almost half of the mean cost for HE-DMTs), AHSCT could offer further advantages as it may induce higher rates of disability improvement compared to HE-DMTs [30]. The latter effect may contribute to additional savings of the indirect costs related to loss of working abilities and social care expenditure: compared to mild disability, total costs for moderate and severe disability were recently estimated as 1.4–2.3-fold and 1.8–2.9-fold higher, respectively [37].

Our results are consistent with recently published studies. Burt et al. concluded that AHSCT in RR-MS may be a “win–win” in terms of both cost and clinical efficacy, possibly capable of generating cost-savings and additional health gains for well-selected RR-MS patients compared with standard DMTs [6]. In the study by Orlewska et al., AHSCT reduced all treatment-costs by 82%, paying off its costs within 3.9 years after the procedure [7].

The cost-effectiveness of AHSCT in SP-MS is probably less remarkable than in RR-MS. A previous study on cases with moderate-severe disability showed that AHSCT was cost-effective compared to mitoxantrone in 2 out of 3 scenarios evaluated [5], being dominated by the comparator when the duration of the effect was assumed to be sustained for 5 years only.

Despite suggestion for high benefits and cost-savings, the safety profile of AHSCT should not be overlooked: AHSCT should be reserved for highly selected cases with aggressive MS, and it should be performed only in highly specialised and accredited centres in order to reduce periprocedural risks.

This study has several limitations. First of all, indirect costs due to disease reactivation and disability (including reduction/loss of working abilities) were not included, as well as those due to work absenteeism during the AHSCT procedure. Nonetheless, the latter might be counterbalanced by an improvement in working abilities after AHSCT compared to the pre-treatment status, as previously reported [38]. Costs of treatment-related complications requiring specific management were not considered, as they could not be accurately estimated; such costs may impact mostly on expenses for AHSCT rather than on those for HE-DMTs. In the first 6 months (and up to 1 year) after AHSCT, EBV/CMV reactivation or other infections are relatively frequent and may require different diagnostic/therapeutic work-up, including pre-emptive treatment with rituximab (in case of EBV reactivation), and even hospital re-admission in case of severe infections. As a consequence, “standard costs” could underestimate the real costs of AHSCT, especially if a severe complication occurred. Lastly, the comparison of CNT should be taken with caution as it is based on indirect comparisons of studies including heterogeneous patient populations. Furthermore, estimates at year 5 were available only for a subset of HE-DMTs, limiting further the comparison.

## Conclusions

AHSCT may represent a cost-effective strategy in highly active RR-MS, with remarkable cost-savings compared to HE-DMTs over the long term. Besides priceless benefits for the well-being of AHSCT-treated patients, the use of AHSCT in highly selected cases may produce economic savings that may contribute to improving healthcare assistance at a population level. Such observation may be particularly relevant for low-income countries, where access to HE-DMTs is currently limited.

## Data Availability

Aggregated data will be shared upon written request to the corresponding author.
